# Oral Vaccination against *Lawsonia* *intracellularis* Changes the Intestinal Microbiome in Weaned Piglets

**DOI:** 10.3390/ani11072082

**Published:** 2021-07-13

**Authors:** Robin B. Guevarra, Jae Hyoung Cho, Jin Ho Cho, Jun Hyung Lee, Hyeri Kim, Sheena Kim, Eun Sol Kim, Gi Beom Keum, Suphot Watthanaphansak, Minho Song, Hyeun Bum Kim

**Affiliations:** 1 Department of Animal Resources Science, Dankook University, Cheonan 311-16, Korea; robin.becina.guevarra@gmail.com (R.B.G.); jhcho5216@gmail.com (J.H.C.); sniper9560@naver.com (J.H.L.); hyely26@gmail.com (H.K.); sheen915@gmail.com (S.K.); essol0430@gmail.com (E.S.K.); keumgb@gmail.com (G.B.K.); 2 Division of Food and Animal Sciences, Chungbuk National University, Cheongju 286-44, Korea; jinhcho@cbnu.ac.kr; 3 Departments of Veterinary Medicine, Faculty of Veterinary Science, Chulalongkorn University, Bangkok 10330, Thailand; Supot.W@chula.ac.th; 4 Division of Animal and Dairy Science, Chungnam National University, Daejeon 341-34, Korea

**Keywords:** gut health, gut microbiome, *Lawsonia intracellularis*, pigs

## Abstract

**Simple Summary:**

*Lawsoniaintracellularis* is an obligately intracellular enteric bacterium that infects intestinal epithelial cells causing porcine proliferative enteropathy, which is responsible for economic losses in the swine industry worldwide. This study evaluated the effects of a commercial, oral attenuated *L. intracellularis* vaccination (Enterisol^®^ Ileitis) on the intestinal microbial community of weaned piglets. Piglets were experimentally vaccinated with different doses of the *L*. *intracellularis* vaccine, and the gut microbial shifts were measured at weeks 0 and 6 post-vaccination. The data presented here demonstrated that oral vaccination against *L*. *intracellularis* shapes the gut microbiota in weaned piglets. Alpha diversity analysis revealed that vaccination led to significant changes in species evenness but no changes in species richness of the gut microbiota. Beta diversity analysis revealed that vaccination against *L. intracellularis* caused a shift in the microbial community structure. At the phylum level, Firmicutes and Bacteroidetes were the most abundant phyla in the intestinal microbiota of piglets regardless of treatment group and time points. At the genus level, there was a significant increase in *Streptococcus* and a significant decrease in *Clostridium* in the fecal microbiota of vaccinated pigs, regardless of the dose. Overall, this study sheds a new light on the potential role of the pig microbiota in modulating vaccine responses.

**Abstract:**

*Lawsoniaintracellularis*, which causes porcine proliferative enteropathy (PPE), is a common swine intestinal pathogen that is prevalent in pig production sites worldwide. In this study, the alteration in the microbiome composition of weaned pigs was investigated in response to vaccination against *L. intracellularis,* using 16S rRNA gene sequencing. A total of 64 crossbred (Duroc × [Landrace × Yorkshire]) healthy weanling pigs weaned at 4 weeks of age were randomly assigned to four treatment groups (four pigs/pen; four pens/treatment), using a randomized complete block design for the 42-day trial. Pigs in the treatment groups were orally administered with three different doses (1 dose = 2 mL) of vaccine against *L. intracellularis* (Enterisol^®^ Ileitis, Boehringer Ingelheim Vetmedica GmbH), namely the following: LAW1 (0.5 dose), LAW2 (1 dose), LAW3 (2 dose). A non-vaccinated group served as a negative control (CONT). Alpha diversity analysis revealed that vaccination led to significant changes in species evenness but not species richness of the gut microbiota. Beta diversity analysis revealed that vaccination against *L. intracellularis* caused a significant shift in the microbial community structure. At the genus level, there was a significant increase in *Streptococcus* and a significant decrease in *Clostridium* in the fecal microbiota of vaccinated pigs, regardless of dose.

## 1. Introduction

Porcine proliferative enteropathy (PPE), commonly referred to as ileitis, is caused by Gram-negative anaerobic obligate intracellular bacterium *Lawsonia intracellularis,* which is an economically important swine disease that poses significant threats to the swine industry worldwide [1,2]. PPE disease is commonly observed in post-weaned and growing pigs less than 4 months of age and characterized by acute diarrhea, weakness, decreased weight gain, intestinal hemorrhage, and sudden death [3]. The mode of transmission of *L*. *intracellularis* is via the fecal–oral route, and lesions are characterized by thickening of the mucosa of the ileum and colon [2,4]. To date, prophylaxis against *L*. *intracellularis* infection is achieved by either administration of antibiotic growth promoters or vaccination with live attenuated *L*. *intracellularis* vaccine [5]. In a recent study, *Salmonella*-based *L*. *intracellularis* vaccines induced *L*. *intracellularis*-specific humoral and cell mediated immunities and conferred dual protection against PPE and salmonellosis in a murine model [6].

In recent years, considerable research has revealed the importance of the gut microbiome in the health and growth of animals. The intestinal microbiome plays a crucial role in the development and regulation of the immune system and therefore, its composition may affect how animals respond to vaccinations [7]. It has been recognized that the intestinal microbiome affects the immune response to natural infection or vaccination [8]. For example, Leite et al. revealed that vaccination against *L*. *intracellularis* decreased levels of *S. enterica* serovar Typhimurium shedding in co-infected pigs, indicating that vaccination against *L*. *intracellularis* may be used as a tool to prevent foodborne diseases associated with *Salmonella* [9]. *L*. *intracellularis* seroconversion has been identified as a risk factor for the increased prevalence of pigs shedding *Salmonella enterica* subsp. *enterica* in farrow-to-finish herds [10].

However, information regarding the effects of oral vaccination with live attenuated vaccine against *L. intracellularis* in the intestinal microbial composition of pigs is limited. Furthermore, the changes in the intestinal microbiota composition in response to vaccination against *L*. *intracellularis* are not fully understood. This study was performed to determine the effects of oral vaccination against *L. intracellularis* on the intestinal microbiome of weaned piglets. To do this, pigs were experimentally vaccinated with different doses of *L*. *intracellularis* vaccine; their microbial community composition was measured before and after oral administration; and finally, the microbiota of vaccinated pigs were compared to those of non-vaccinated pigs. We hypothesized that different doses of live attenuated vaccine against *L*. *intracellularis* would cause alteration in the composition of the pig gut microbiome. Understanding the impact that the pig gut microbiome plays in response to vaccination will improve our current understanding of vaccine efficacy and the underlying changes in the composition of the microbiome that may be associated with the susceptibility of pigs to PPE. The microbial biomarkers identified in this study may be used for the development of strategies to improve vaccine efficacy against PPE, especially in pigs at early life when the intestinal microbiome is more susceptible to perturbation and plays a crucial role in immune development.

## 2. Materials and Methods

### 2.1. Animals, Housing and Experimental Design

All the piglets used in this experiment were provided by the Chungnam Livestock Research Institute of South Korea (Chungcheongnam-do, South Korea), where pigs are raised free of important swine diseases, including porcine reproductive and respiratory syndrome, foot and mouth disease, and African swine fever. The piglets showed no signs of enteric or respiratory diseases when the animals were obtained for this study. A total of 64 crossbred (Duroc × [Landrace × Yorkshire]) healthy weanling pigs weaned at 4 weeks of age were housed at the Animal Research Center of Chungnam National University, South Korea. Pigs at four weeks of age were randomly divided into four groups comprising 16 animals divided into four pens with 4 pigs per pen, using a randomized complete block design for the 42-day trial. Prior to vaccination, the pigs were allowed to acclimatize for 1 week after weaning at 4 weeks of age. Then, pigs at week 0 (5 weeks of age) in the treatment groups were orally administered with three different doses (1 dose = 2 mL) of vaccine against *L*. *intracellularis* (Enterisol^®^ Ileitis, Boehringer Ingelheim Vetmedica, Inc., Ingelheim am Rhein, Germany), namely, LAW1 (0.5 dose), LAW2 (1 dose), and LAW3 (2 dose). The *L*. *intracellularis* vaccine was prepared by rehydrating the lyophilized live attenuated bacteria supplied as a solid cake with a solution of liquid diluent, according to the manufacturer’s instructions, and administered directly by oral drench to each pig. The pigs in pen number 1 remained unvaccinated and served as controls (CONT). All pigs in this study had similar husbandry practices to the Animal Research Center of Chungnam National University, South Korea. Room temperature was maintained at 26 °C, and the humidity was maintained constant at 60%. The pigs were housed in pens with totally slotted floors. Each pen was installed with a one-sided self-feeder and a nipple water-feeder, and pigs were given ad libitum access to feed and water throughout the experiment. A wheat–soybean meal basal diet was formulated to meet the nutrient requirements as suggested by National Research Council (NRC) [11]. All the pigs were fed the basal diet without any antibiotics or supplementary additives. The experimental procedures used in this study were performed in accordance with the recommendations put forth in the Guide for the Use and Care of Laboratory Animals, and were approved by the Animal Care and Use Committee of Chungnam National University. Humane animal care was practiced throughout the trial and every effort was made to minimize suffering for the piglets.

### 2.2. Sample Collection and Genomic DNA Extraction

A total of 123 fresh fecal samples were collected directly from the rectum of each animal at week 0 (5 weeks of age, *n* = 63) and week 6 (11 weeks of age, *n* = 60) of the experiment after oral administration of the *L*. *intracellularis* vaccine. We were not able to collect a fecal sample from one piglet in LAW1 (0.5 dose) group at week 0. Before this experiment ended at week 6, two piglets from each LAW1 group (0.5 dose) and LAW3 group (2 dose) were dead, resulting in 60 samples at week 6. A total of 200 mg of feces per sample was used to extract the total DNA representing the pig gut microbial communities, using QIAamp Fast DNA Stool Mini Kit (QIAGEN, Hilden, Germany) with minor modifications. Briefly, two rounds of bead-beating the samples for 2 min at 300× *g* was performed with a 5 min incubation in a water bath at 70 °C between beatings. The Colibri Microvolume Spectrometer (Titertek Berthold, Pforzheim, Germany) was used to measure the concentrations of DNA, and DNAs with OD260/280 ratios of 1.80–2.15 were used in this study.

### 2.3. 16S rRNA Gene PCR

The V5 to V6 hypervariable regions of the 16S rRNA genes were amplified, using the universal PCR primers 799F-mod6 (5′ CMGGATTAGATACCCKGGT-3′) and 1114R (5′-GGGTTGCGCTCGTTGC-3′). The PCR amplification mixture in a reaction volume of 50 µL contained 10 pmol of each primer, 25 ng of DNA, 2.5 mM concentrations of each deoxynucleotide triphosphates (dNTPs), 2.5 U/µL of PrimeSTAR HS DNA Polymerase, and 5X PrimeSTAR Buffer (Mg^2+^) (Takara Bio, Inc., Shiga, Japan). The PCR cycling parameters included an initial 3 min denaturation at 98 °C, followed by 30 cycles of 98 °C for 10 s, 55 °C for 15 s, and 72 °C for 30 s, and a final 3 min extension at 72 °C. The Wizard^®^ SV Gel and PCR Clean-Up System purification kit (Promega, Madison, WI, USA) were used to purify the PCR products.

### 2.4. 16S rRNA Gene Library Preparation and MiSeq Sequencing

The Illumina MiSeq platform at Macrogen Inc. (Seoul, Korea) was used to sequence 16S rRNA gene amplicons. Briefly, random fragmentation of the DNA samples followed by 5′ and 3′ adapter ligation was conducted to prepare the sequencing library. During this step, dual indices and Illumina sequencing adapters were attached to the 16S rRNA gene amplicons, using the Nextera XT Index Kit (Illumina, San Diego, CA, USA). The concentration of final products was normalized and pooled, using PicoGreen (Turner BioSystems, Inc., Sunnyvale, CA, USA), then the verification of the size of libraries was conducted using the TapeStation DNA ScreenTape D1000 (Agilent Technologies, Inc., Santa Clara, CA, USA). The PCR cycle conditions included a 3 min initial denaturation at 95 °C, 8 amplification cycles (95 °C for 30 s, 55 °C for 30 s, and 72 °C for 30 s) and a 5 min final elongation at 72 °C.

### 2.5. 16S rRNA Gene Analysis

The quality control of the raw sequence data generated from the Illumina MiSeq platform was performed. The sequences with a length of less than 200 bp and containing ambiguous base calls were eliminated from the demultiplexed sequence reads to minimize the effects of random sequencing errors. The UCHIME algorithm implemented in Mothur was used to identify the chimeric sequences, and those chimeric sequences were excluded for downstream analysis. Then, the operational taxonomic unit (OTU) picking using the open-reference OTU picking workflow with the SortMeRNA and SUMACLUST methods for reference OTU and de novo OTU picking were conducted, using the QIIME (Quantitative Insights into Microbial Ecology) pipeline (version 1.9.1) [12]. The naïve Bayesian Ribosomal Database Project (RDP) classifier based on GreenGenes taxonomy reference database version 13_8 was used for the taxonomic assignment of sequence reads. For the downstream analysis, low-abundance OTUs and singletons were eliminated from the OTU table with minimum count of 4 and low-count filter based on 20% prevalence in the samples. Then, normalization of the data was conducted by rarefying the data to the minimum library size and by data scaling, using the total sum scaling (TSS) before any statistical comparison to address the sparsity of the data and the variability in the sampling depth.

### 2.6. Statistical Analysis

The R package MicrobiomeAnalystR and GraphPad Prism v7.00 (La Jolla, CA, USA) was used to conduct the statistical analysis. The MicrobiomeAnalystR was used to compute alpha diversity measures, including Chao1, observed OTUs, Shannon and Simpson diversity indices. Comparison of alpha diversity among the groups was conducted using the non-parametric Kruskal–Wallis test. The significant difference level was set at *p* < 0.05. The principal coordinate analysis (PCoA) plots at the OTU level were generated, using the weighted and unweighted UniFrac distances. Comparison of beta-diversity among the groups was conducted, using the Analysis of Similarities (ANOSIM) based on the unweighted and weighted UniFrac distances. The default parameters at 20% sample prevalence and 0.2% relative abundance were used to generate the heatmap of the core microbiota. The Euclidean distance measure and Ward clustering algorithm were used to generate the heatmap of hierarchical clustering. To elucidate the bacterial taxa that are associated with each group, the linear discriminant analysis (LDA) Effect Size (LEfSe) at the OTU level based on the non-parametric Kruskal–Wallis sum–rank test with a log LDA score threshold of 2.0 was used. A *p*-Value < 0.05 was considered significant.

## 3. Results

### 3.1. Animals

During the 42-day feeding trial, pigs showed normal growth pattern and appeared to be healthy regardless of the treatment (Table S1).

### 3.2. Summary of DNA Sequence Data

Total DNA was extracted from fecal samples of the pigs, and the extracted community DNA was PCR amplified and sequenced, using primers specific to the V5 to V6 hypervariable regions of the 16S rRNA genes. Sequencing of the 16S rRNA genes in fecal samples produced a total of 22,590,931 reads, which ranged from 101,498 to 325,783 reads per sample. The average quality score (Phred scores) across all the samples ranged from 32 to 36. Phred scores greater than Q30 indicated that that there was less than 0.1% chance that a base was called incorrectly. Further data filtering was performed in the OTU table to remove low quality or uninformative data and to improve the downstream statistical analysis (Table S2). The OTU table resulted in 7,940,551 combined reads from all the samples, with an average reads per sample of 64,554.

### 3.3. Alpha Diversity

**Figure 1:**
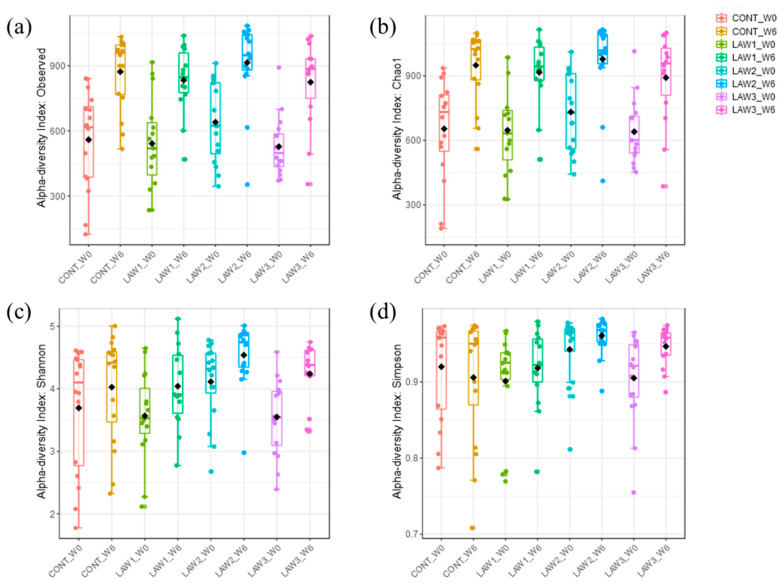
Box plots showing the alpha diversity indices in gut microbiomes of pigs orally vaccinated with *Lawsonia intracellularis* vaccine. Species richness were measured using (**a**) number of observed OTUs and (**b**) Chao1 diversity index. Species evenness and diversity were measured using (**c**) Shannon and (**d**) Simpson diversity indices. Boxes represent the interquartile range (IQR) between the 25th and 75th percentile, and the horizontal line inside the box denotes the median value. Whiskers represent the lowest and highest values within 1.5 times from the 25th and 75th quartiles, respectively. Boxes are colored according to the treatment group and time points as shown in the legend. W0 and W6 denote week 0 and week 6, respectively.

The diversity of the microbial communities in the fecal samples of piglets was measured using the following diversity indices: number of observed OTUs and Chao1, which are species richness estimators, and Shannon and Simpson indices, which take into account species evenness within a sample (Figure 1). At week 0, the number of observed OTUs and Chao1 diversity index ranged from 124–916 to 190–1014, respectively. The Shannon and Simpson values ranged from 1.77–4.78 to 0.76–0.98, respectively, among the four groups. Interestingly, the highest number of observed OTUs, Chao1, Shannon and Simpson values were observed in the LAW2 group. At week 6 post-vaccination, the number of observed OTUs and Chao1 diversity indices among the four groups ranged from 353–1084 to 386.1–1116, respectively. The highest number of observed OTUs and Chao1 at week 6 was observed in the LAW2 group with mean values 914.1 ± 189.9 and 978.4 ± 188.3, respectively, indicating that immunization of 1 dose of *L*. *intracellularis* vaccine increased the number of unique species in the fecal microbial communities in the pigs. The Shannon and Simpson values ranged from 2.32–5.12 to 0.70–0.98, respectively, among the four groups. Similarly, the highest Shannon and Simpson index values at week 6 were observed in the LAW2 group with mean values of 4.54 ± 0.50 and 0.96 ± 0.02, respectively, suggesting that immunization of 1 dose of the *L*. *intracellularis* vaccine also increased the species richness and diversity in the feces of weaned piglets at week 6. However, no statistical differences were observed with Chao1, the number of observed OTUs, and the Shannon index at both week 0 and week 6. Nevertheless, the Simpson index showed significant difference between treatments with increased values in LAW2 and LAW3 as compared to the CONT group (*p* < 0.05) at both week 0 and week 6. Moreover, we compared the alpha diversity between week 0 and week 6 regardless of the treatment group. All four alpha diversity indices (Chao1, observed OTUs, Shannon and Simpson indices) were significantly higher at week 6 than in week 0, suggesting that species richness and diversity increase as the pig age increases (*p* < 0.001).

### 3.4. Beta Diversity

**Figure 2:**
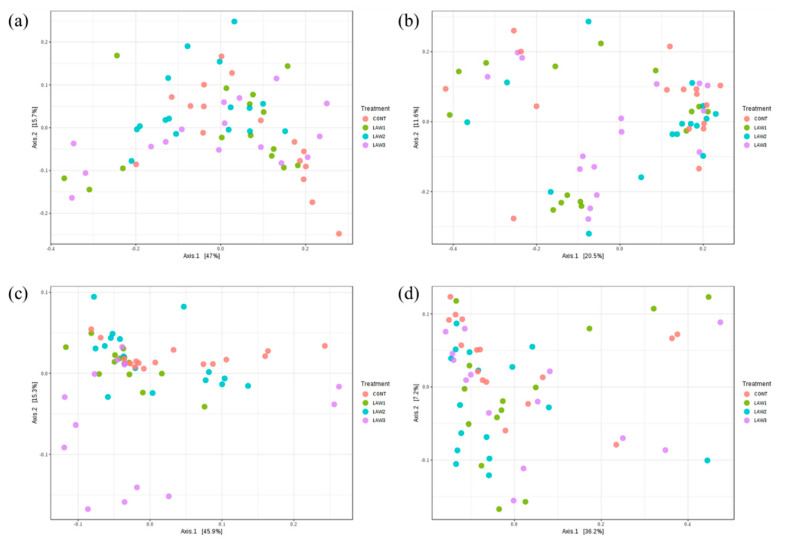
Beta diversity analysis of gut microbiota in piglets orally vaccinated with *Lawsonia intracellularis* vaccine. Principal coordinates analysis (PCoA) plots of pig gut microbiota at week 0 (**a**,**b**) (ANOSIM: R = 0.01, *p* < 0.175) and at week 6 (**c**,**d**) (ANOSIM: R = 0.11, *p* < 0.001) based on the weighted (**a**,**c**) and unweighted (**b**,**d**) UniFrac distances. Dots represent the gut microbiota from each sample and are color coded according to the treatment group (CONT, LAW1, LAW2, and LAW3) as shown in the legend.

At week 0, the PCoA plot shows no significant separation of the microbial community structure among the treatment groups as confirmed by ANOSIM, using both the weighted (Figure 2a) and unweighted (Figure 2b) UniFrac distances (*p* > 0.05). Conversely, at week 6 post-vaccination, the PCoA plot shows that the gut microbiota from the LAW3 group were clustered apart from all the other treatment groups, whereas CONT, LAW1 and LAW2 were clustered closely to each other, based on the weighted UniFrac distances (Figure 2c). Notably, a significant difference in the clustering of samples was observed as confirmed by ANOSIM based on the weighted UniFrac distance (R = 0.11 and *p* < 0.001) (Figure 2c), indicating that a higher dosage of the *L*. *intracellularis* vaccine had a significant impact on the microbial community structure in the weaned piglets. However, no significant difference in microbial community membership was observed among the four treatment groups using the unweighted UniFrac distance (*p* > 0.05) (Figure 2d). Regardless of the treatment, the beta diversity was significantly different between week 0 and week 6 as confirmed by ANOSIM based on the unweighted (*R* = 0.37, *p* < 0.01), and weighted UniFrac distances (*R* = 0.20, *p* < 0.01), indicating that the microbial community structure changes over time.

### 3.5. Microbiome Composition of Pigs Associated with Lawsonia intracellularis Vaccination at the Phylum Level

**Figure 3:**
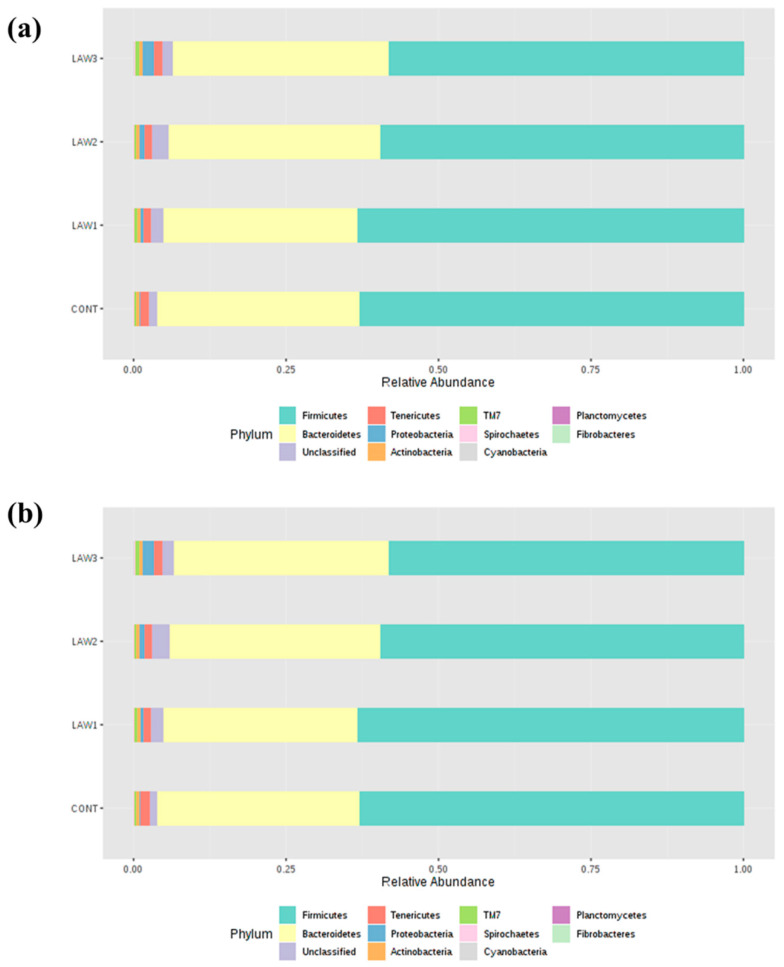
Gut microbiota composition at the phylum level in pigs orally vaccinated with *Lawsonia intracellularis* vaccine. Bar plots show the relative abundance of taxa at the phylum level at (**a**) week 0 and at (**b**) week 6 among the four treatment groups (CONT, LAW1, LAW2 and LAW3).

The intestinal microbiome of pigs vaccinated with different doses of oral vaccine against *L*. *intracellularis* were determined. The relative abundance of different bacterial taxa at the phylum level among the four groups (CONT, LAW1, LAW2, and LAW3) between week 0 and week 6 is shown in Figure 3. The most common phyla in all the samples at both week 0 and week 6 were Firmicutes and Bacteroidetes. At week 0, Firmicutes and Bacteroidetes were the most predominant bacteria, ranging from 82.96 to 94.66% with the highest abundance from the LAW2 group accounting for 94.66% followed by the LAW3 (87.08%), LAW1 (85.91%) and CONT (82.96%) groups. In addition, the relative abundance of Proteobacteria ranged from 0.71 to 11.94% with the highest abundance from the CONT group (11.94%) followed by LAW1 (10.04%), LAW3 (9.52%) and LAW2 (0.71%). At week 6, Firmicutes and Bacteroidetes were also the most predominant phyla in all the samples, which collectively ranged from 93.13 to 96.25% of the total bacteria with the CONT group having the highest combined relative abundance of 96.25% followed by LAW1 (95.13%), LAW2 (94.48%) and LAW3 (93.13%). Moreover, the relative abundance of Proteobacteria ranged from 0.27 to 2.19% of all the bacteria with the lowest and highest in the CONT and LAW3 group, respectively. These findings were similar to previous studies in swine gut microbiota, suggesting that phylum Firmicutes and Bacteroidetes are the major phyla, regardless of *L*. *intracellularis* vaccine treatment.

### 3.6. Microbiome Composition of Pigs Associated with Lawsonia intracellularis Vaccination at the Genus Level

**Figure 4:**
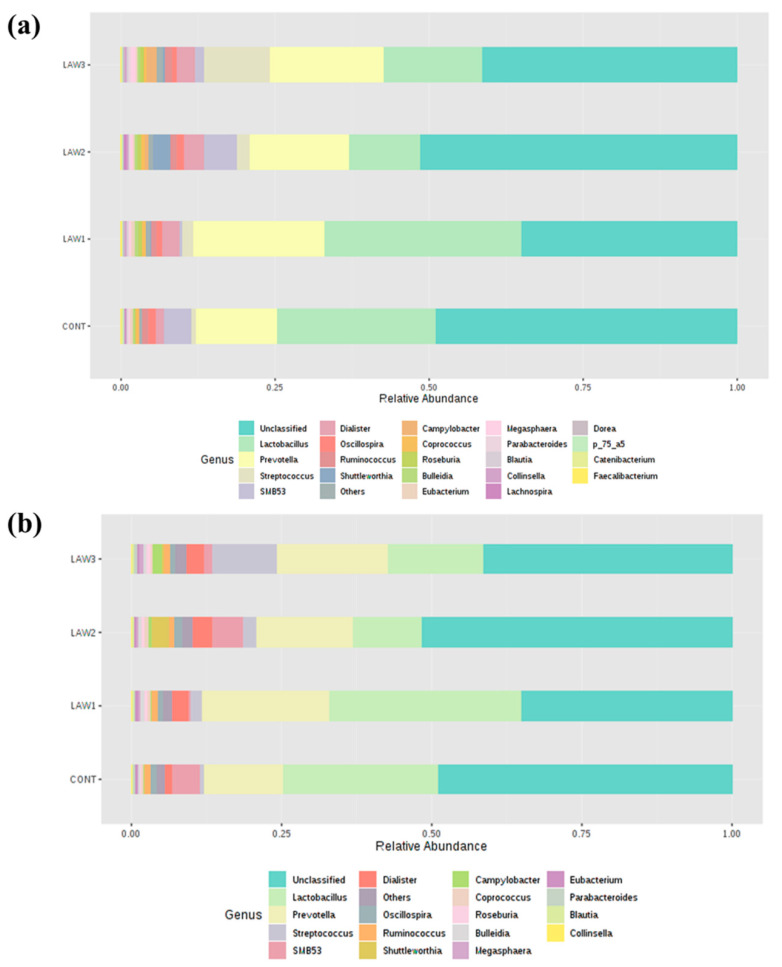
Gut microbiota composition at the genus level in pigs orally vaccinated with *Lawsonia intracellularis* vaccine. Bar plots show the relative abundance of taxa at the genus level at (**a**) week 0 and at (**b**) week 6 among the four treatment groups (CONT, LAW1, LAW2 and LAW3).

The relative abundance of different bacterial taxa at the genus level among the four groups (CONT, LAW1, LAW2, and LAW3) between week 0 and week 6 is shown in Figure 4. The OTUs without taxa destination were collapsed into “Unclassified”. The bacterial genera with counts lower than 5000 were merged into “Others”. Regardless of the time point, the top two most predominant classified genera in the fecal microbiota of pigs orally vaccinated with *L*. *intracellularis* vaccine were *Lactobacillus* and *Prevotella*. At week 0, the relative abundance of *Lactobacillus* ranged from 15.77 to 23.77% with the highest and lowest abundance detected in the LAW3 and LAW2 groups, respectively. The genus *Prevotella* was found as most predominant in the LAW2 group (14.24%) and was found to be the lowest in the CONT group (3.76%). Notably, most of the bacteria were “Unclassified”, which accounts for 57.21–63.66% of the total reads in each group at week 0.

Similarly, at week 6, *Lactobacillus* and *Prevotella* were the most predominant bacterial genera, which collectively accounted for 27.76–54.84% of the total reads in all samples. The highest and lowest relative abundance of *Lactobacillus* was found in LAW1 (33.39%) and LAW2 (11.10%) group, respectively. In contrast, the relative abundance of *Prevotella* was highest in LAW1 (21.45%) and lowest in the CONT (13.12%) group. Moreover, the relative abundance of “Unclassified” OTUs ranged from 32.95 to 51.52% of the total bacteria in all groups, with the lowest and highest percentage abundance from the LAW1 and LAW2 groups, respectively.

### 3.7. Microbial Shifts in the Fecal Microbiota of Pigs Associated with Lawsonia intracellularis Vaccination

**Figure 5:**
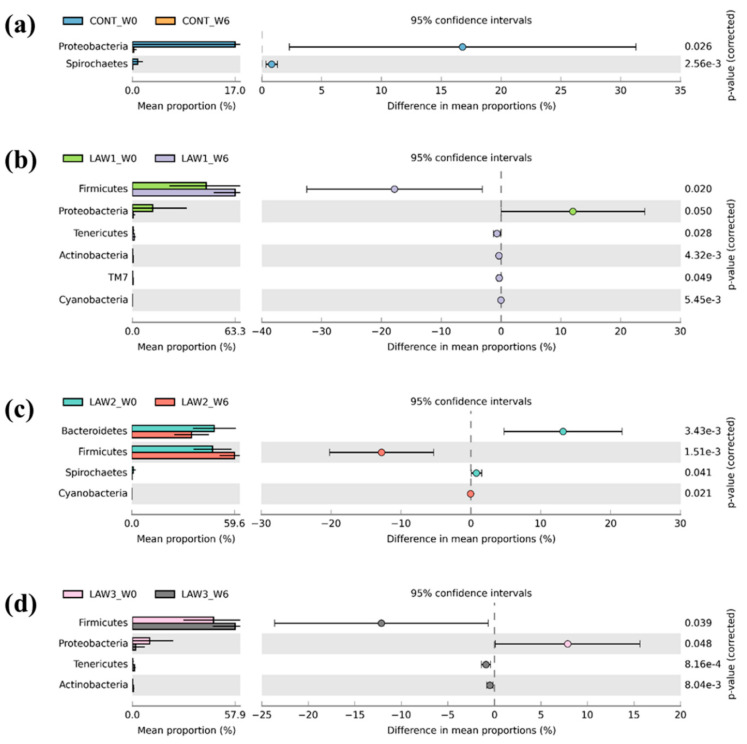
Extended error plots identifying significantly different taxa at the phylum level in pigs orally vaccinated with *Lawsonia intracellularis* vaccine. Significant differential abundance of phyla between week 0 (W0) and week 6 (W6) for each of the treatment groups (**a**) CONT, (**b**) LAW1, (**c**) LAW2, and (**d**) LAW3 are illustrated. Corrected *p*-Values are shown at the right. Statistical significance is measured using a two-sided Welch’s *t*-test and a *p* < 0.05 is considered significant.

**Figure 6:**
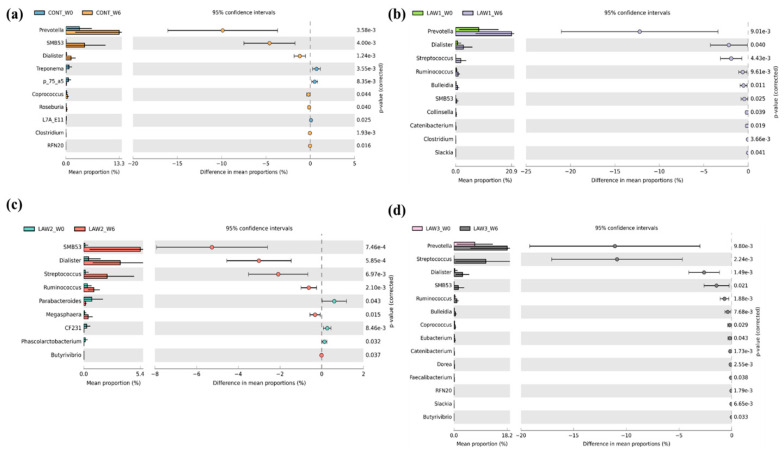
Extended error plots identifying significantly different taxa at the genus level associated with *Lawsonia intracellularis* vaccination. Significant differential abundance of phyla between week 0 (W0) and week 6 (W6) for each of the treatment groups (**a**) CONT, (**b**) LAW1, (**c**) LAW2, and (**d**) LAW3 are illustrated. Corrected *p*-Values are shown at the right. Statistical significance is measured using a two-sided Welch’s t-test and a *p* < 0.05 is considered significant.

We compared the differences in the relative abundance of taxa at the phylum and genus levels between week 0 and week 6 in each treatment group, using the two-sided Welch’s *t*-test in STAMP, and visualized them, using an extended error plot (Figure 5 and Figure 6). At the phylum level, there was a significant decrease in the population of Proteobacteria and Spirochaetes (*p* < 0.05) in the non-vaccinated CONT group (Figure 5). In the LAW1 group, the population of phyla Firmicutes, Tenericutes, Actinobacteria, TM7 and Cyanobacteria were significantly increased, whereas the population of Proteobacteria was significantly decreased (*p* < 0.05). In the LAW2 group, the relative abundance of phyla Firmicutes and Cyanobacteria were significantly increased, while members of phyla Bacteroidetes and Spirochaetes were significantly decreased (*p* < 0.05). In the LAW3 group, the relative abundance of Firmicutes, Tenericutes and Actinobacteria were significantly increased, whereas the population of Proteobacteria was significantly decreased (*p* < 0.05) (Figure 5).

At the genus level, the differences in mean proportions of taxa and the statistical significance are shown in Figure 6. Notably, the population of *Prevotella* was significantly increased in all the groups, except for the LAW2 group. The relative abundance of SMB53 and *Dialister* were significantly enriched in all the groups regardless of treatment. The relative abundance of *Butyrivibrio* was significantly increased in the LAW2 and LAW3 groups. Interestingly, the relative abundance of *Streptococcus* and *Ruminococcus* were significantly enriched in all pigs vaccinated with *L*. *intracellularis* vaccine regardless of dose, except in the CONT group (*p* < 0.001). Moreover, the *Streptococcus* populations in the intestinal microbiota of piglets increased as the dose of *L*. *intracellularis* vaccine increased.

### 3.8. Differentially Abundant Genera Associated with Oral Vaccination against L. intracellularis in Weaned Piglets

**Figure 7:**
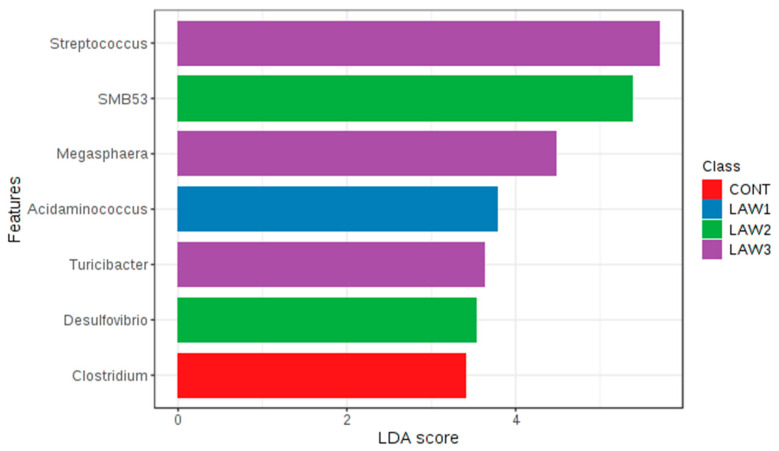
Differentially abundant genera in the pig gut microbiota among the four treatment groups orally vaccinated with *Lawsonia intracellularis* vaccine at week 6. The linear discriminant analysis (LDA) effect size (LEfSe) algorithm is used for biomarker discovery, which emphasizes both statistical and biological relevance. A *p*-Value of <0.05 is considered significant in Kruskal–Wallis and pairwise Wilcoxon tests, respectively. The LDA score for discriminative features is 3. The length of the histogram represents the LDA score, which explains the degree of influence of species with significant difference among the four treatment groups (CONT, LAW1, LAW2, and LAW3).

**Figure 8:**
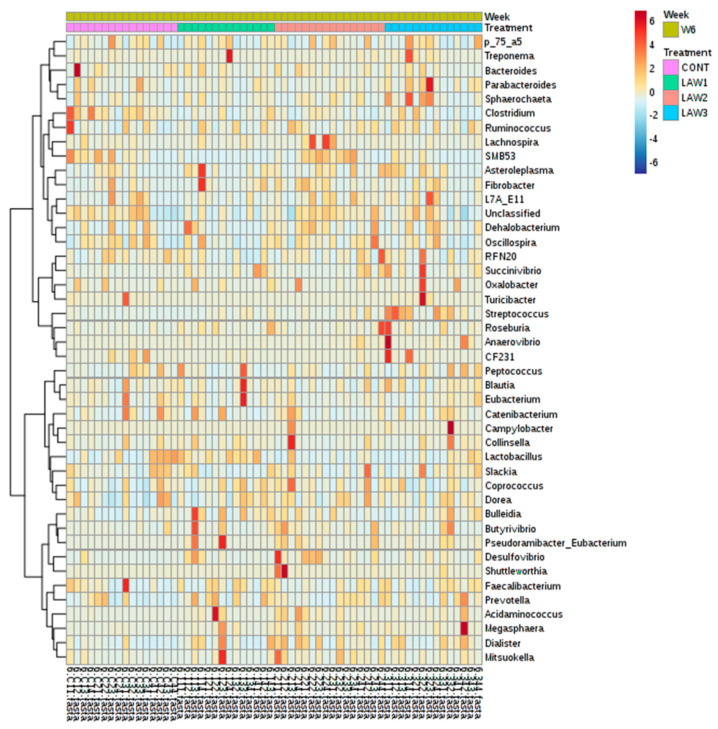
Hierarchical clustering heatmap of bacterial genera among the treatment groups (CONT, LAW1, LAW2, and LAW3) at week 6 based on Euclidean distance measure and Ward clustering algorithm.

Comparable enrichment analysis of species by linear discriminant analysis (LDA) revealed that some bacterial species were unique biomarkers to the intestinal microbiome of the pig groups experimentally vaccinated with different doses of oral vaccine against *L*. *intracellularis*. The LDA effect size (LEfSe) based on the non-parametric Kruskal–Wallis sum–rank test was used to identify significantly enriched genera in each treatment group at week 6 (Figure 7). At week 0, no significant taxa were detected among the treatment groups at the genus level using LEfSe analysis and MetagenomeSeq (*p* > 0.05). At week 6, the LEfSe analysis showed that the LAW1 group had significantly the highest relative abundance of *Acidaminococcus* among the four groups. In addition, genera *SMB53* and *Desulfovibrio* were significantly enriched in the LAW2 group. In addition, the LAW3 group were significantly enriched in the genera *Streptococcus*, *Megasphaera* and *Turicibacter*. Moreover, the MetagenomeSeq analysis revealed that *Clostridium* and *Turicibacter* were significantly lower in three pig groups experimentally vaccinated with *L*. *intracellularis* as compared to the non-vaccinated CONT group (*p* < 0.05). For better visualization of differentially abundant bacterial genera among the treatment groups, a hierarchical clustering heat map was created as shown in Figure 8.

### 3.9. Core Microbiome of Weaned Pigs Associated with Lawsonia intracellularis Vaccination

**Figure 9:**
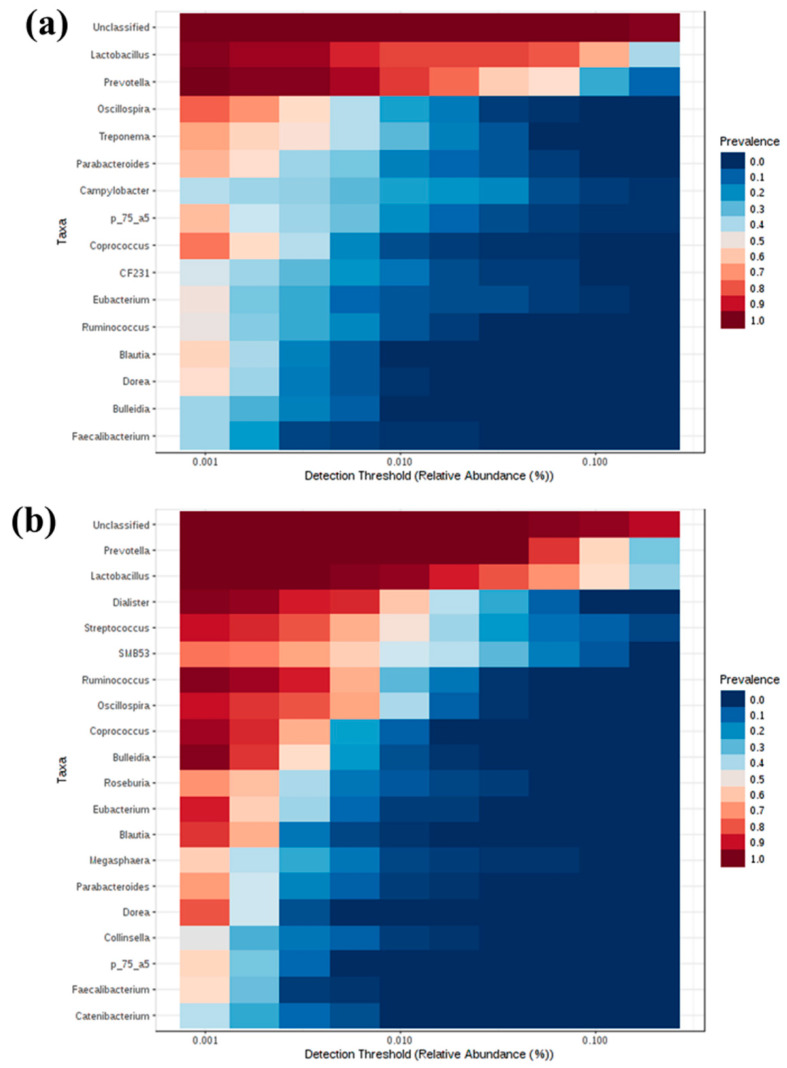
Core microbiota in pigs orally vaccinated with *Lawsonia intracellularis* vaccine. Heatmap depicting the core OTUs and their prevalence at different detection thresholds at (**a**) week 0 and (**b**) week (6).

Core microbiome analysis was performed at the genus level based on the sample prevalence and relative abundance at a cut-off value of 20% and 0.2%, respectively. The core microbiome consisted of OTUs common to all samples from each individual pig. At week 0, 16 bacterial genera were identified as the core microbiome and the top five most prevalent genera were *Lactobacillus*, *Prevotella*, *Oscillospira*, *Treponema* and *Parabacteroides* (Figure 9a). At week 6, 18 core bacterial genera were identified, and the top 5 most prevalent core microbiota were *Prevotella*, *Lactobacillus*, *Dialister*, *Streptococcus* and SMB53 (Figure 9b).

## 4. Discussion

This study was performed to determine the effects of oral vaccination against *L*. *intracellularis* on the intestinal microbiome in weaned piglets. To do this, pigs were experimentally vaccinated with different doses of *L*. *intracellularis* vaccine and the differences in the microbial composition between two time points at week 0 and week 6 post vaccination were measured in order to determine the shifts in the intestinal microbiota.

The work presented here demonstrated alterations in the composition of the gut microbiomes of pigs experimentally vaccinated with different doses of live attenuated vaccine against *L*. *intracellularis*. Alpha diversity analysis revealed that vaccination against *L*. *intracellularis* led to a significant increase in the Simpson diversity index, suggesting that significant changes in the alpha diversity were only observed in measures of species evenness and not species richness in pigs. However, no statistical differences were observed with Chao1, the number of observed OTUs, or the Shannon index at both week 0 and week 6. This also indicates that the number of species in the intestine of pigs was not altered as much as their quantities, as influenced by oral vaccination against *L*. *intracellularis* in weaned piglets.

Moreover, previous studies showed that oral challenge with *L*. *intracellularis* led to changes in the composition of the swine microbiome [13]. In this study, we determined whether oral vaccination with different doses of live attenuated vaccine against *L*. *intracellularis* would change the microbial community structure in weaned piglets. Beta-diversity was measured using the weighted UniFrac distance, which considers the relative abundance of OTUs in the community, and the unweighted UniFrac distance, which takes into account the community membership (presence or absence of OTUs) [14]. The weighted and unweighted UniFrac distances were used to perform PCoA analysis to evaluate the community structure differences among the various treatment groups over the two time points. In this study, the overall compositions of the microbiomes were significantly separated by different doses of *L*. *intracellularis* vaccination with LAW 3 clustered distinctly, indicating that a higher dosage of *L*. *intracellularis* vaccine could induce alterations of the pig gut microbial community in weaned piglets. In addition, clustering of the microbiome compositions in non-vaccinated controls were segregated in different parts of the PCoA. However, the PCoA plot based on the unweighted UniFrac showed that all pigs had a similar microbiome composition regardless of the vaccination treatment (*p* > 0.05). Meanwhile, microbial community structures were significantly different regardless of *L*. *intracellularis* vaccination between week 0 and week 6 (*p* < 0.01), indicating that the intestinal microbiome of the pigs changed as the pigs aged, with an acquired microbial community composition similar to that of the adult pig gut microbiota.

We then analyzed the beta diversity separately at each time point to investigate only the effects of *L*. *intracellularis* vaccination on the microbial community structure among the treatment groups. At week 0, no significant differences in beta diversity were observed, suggesting that *L*. *intracellularis* vaccination did not have a significant impact on the microbial community structure and membership at the initial time point of the study. At week 6, it was possible to observe that *L*. *intracellularis* vaccination in pigs had a significant impact on the microbial community structure in pigs with a higher vaccine dose, which led to increased significant separation of the microbiota in comparison to the non-vaccinated pigs. Our findings were similar to the observations of Leite et al. [9]; however, the advantage of this study was that significant differences observed in microbial community structure and composition were clearly due to *L*. *intracellularis* vaccination since we did not dually infect the pigs with other bacteria, such as *S*. *enterica*. Overall, our results indicate that the overall composition and structure of the pig gut microbiome was altered by oral vaccination against *L*. *intracellularis*.

To further understand how the oral vaccination of *L*. *intracellularis* mediated the microbiome shifts in weaned piglets, we looked for differences in the relative abundance of differentially abundant taxa at the phylum and genus level among the treatment groups. It is known that *L*. *intracellularis* infection can lead to changes in the intestinal microbiome of pigs, indicating complex association. In this study, taxonomic analysis at the phylum revealed that Firmicutes and Bacteroidetes were the most abundant phyla in the intestinal microbiota of piglets regardless of treatment group and time points, which accounted for more than 80% of the bacterial community, in line with other studies on pig gut microbiota [15,16]. Strikingly, a noticeable finding of this study was a significantly higher relative abundance of phylum Firmicutes by oral administration of *L*. *intracellularis* vaccine, regardless of the dose as compared to non-vaccinated controls. This finding is similar to those obtained by Leite et al. [9], which showed higher abundance of *Firmicutes* in the pig intestinal microbiome in response to infection with *L*. *intracellularis*. Conventionally, in piglets, the relative abundance of Firmicutes decreases and that of Bacteroidetes increases with an increase in age [17]. Moreover, it is known that the gut microbiota of pigs early in life is more amenable to changes, hence it can be speculated that alteration in the composition of the microbiome observed in this study may be associated with oral vaccination against *L*. *intracellularis*.

At the genus level, one of the most striking observations in this study was the significant increase in *Streptococcus* and significant decrease in *Clostridium* in the fecal microbiota of pigs that received oral vaccination against *L*. *intracellularis* regardless of dose. Interestingly, our results are similar with those of Borewicz et al., who reported that *L*. *intracellularis* infection could increase the level of *Streptococcus* in the jejunal and ileal tissues of pigs vaccinated at 9 weeks of age [13]. These findings indicate that similar changes in the gut microbiome were observed whether the pigs were experimentally vaccinated with a live attenuated vaccine or naturally infected with *L*. *intracellularis* [13]. Previous studies revealed that some members of *Streptococcus* are potentially harmful and beneficial in the intestine of pigs. For example, *Streptococcus suis* is a notable pathogen in the pig industry, causing a wide variety of diseases, including meningitis, septicemia and many other infections [18]. However, it is uncertain whether the *Streptococcus* observed in this study is a harmful or beneficial bacteria. Moreover, we observed a significant decrease in the abundance of the *Clostridium* species in the intestinal microbiota of pigs that received oral vaccination of *L*. *intracellularis*. In a similar study, the relative abundance of *Clostridium butyricum* was significantly decreased in pigs dually vaccinated with *S*. *enterica* and *L*. *intracellularis* [9]. Some *Clostridium* spp. have been shown to modulate the colonic luminal metabolome by producing short-chain fatty acids, including butyrate, which can help in maintaining the gut health [19]. Butyrate and its derivatives are reported to have positive effects on animal production, including the control of enteric pathogens, reduction in inflammation and modulation of gut microbiota [20]. Therefore, depletion of the *Clostridium* species abundance could promote and benefit the growth of pathobiont species in the pig intestinal microbiota. On the other hand, we also can say that the depletion of the *Clostridium* species could also benefit pig health because some *Clostridium* species are classified as pathogens. Overall, our results suggest that the significant increase in the abundance of *Streptococcus* and decrease in *Clostridium* due to *L*. *intracellularis* vaccination may play a role in maintaining pig health; however, further studies are required to determine how *L*. *intracellularis* vaccination affects piglet performance and gut health through pig gut microbiome modulation.

To date, very few studies have evaluated the effects of vaccination on the microbiome in pigs. Meanwhile, in human gut microbiota studies, colonization by specific bacteria, such as *Bifidobacterium,* may improve vaccine response later in life, while other bacteria may lower vaccine response due to intestinal dysbiosis [21,22]. Previous studies provided clear evidence that the gut microbiota plays an important role in driving host health and vaccine response in pigs. For example, Munyaka et al. investigated the early-life fecal microbiota and transcriptome profiles in whole blood after vaccination for *Mycoplasma hyopneumoniae* and revealed that gene expression and gut microbiota profiles could predict the vaccine response in piglets [23]. In addition, a similar study by Leite et al. indicated that vaccination against *L*. *intracellularis* may be used as a promising tool to prevent *S*. *enterica* in swine production [9]. In this study, some light has been shed into the potential contribution of early-life gut microbiota in the modulation of health and disease in response to vaccination against *L*. *intracellularis* in weaned piglets.

## 5. Conclusions

In summary, the data presented here demonstrate that vaccination against *L*. *intracellularis* could cause disruptions in the pig gut microbiota. We also revealed that administration of different doses of *L*. *intracellularis* vaccine cause differential changes in the gut microbiota over time. Although this study has provided details of the microbial community structure in the pig intestine in response to *L*. *intracellularis* vaccination, understanding of the pig immune response should be taken into account for future studies. The pig gut microbiota plays an important role in immune modulation and may impact the immune response to vaccination against *L*. *intracellularis*. The use of vaccination against *L*. *intracellularis* to control porcine proliferative enteropathy significantly enriched the population of *Streptococcus* and depletion of *Clostridium* species, which may provide benefits and growth of potential pathogens. The findings of this study should be confirmed with evidence-based experiments. Furthermore, it is important to look at the functional characteristics and metabolites of important microbes identified in this study through shotgun metagenomics and metabolomics to obtain a better picture of host-microbiota interactions and the interplay with the *L*. *intracellularis* vaccine. This study highlights areas where the pig gut microbiome holds specific promise in vaccinology.

## Data Availability

All raw 16S rRNA gene data used in this study were deposited in the National Center for Biotechnology Information (NCBI) under BioProject accession number PRJNA576704.

## Supplementary Materials

The following are available online at https://www.mdpi.com/article/10.3390/ani11072082/s1, **Table S1**: Effects of *L. intracellularis* vaccine on growth performance in weaned piglets. **Table S2**: Number of 16S rRNA gene sequences before and after quality control in pigs orally vaccinated with *L. intracellularis* vaccine at week 0 and week 6. Reference [24] is cited in the supplementary material.
