# Supplementary material for: Oral Vaccination against Lawsonia intracellularis Changes the Intestinal Microbiome in Weaned Piglets
Source: Animals (Basel). 2021 Jul 13;11(7):2082. doi: 10.3390/ani11072082 (PMC8300401; doi:10.3390/ani11072082)
Supplement: Supplementary file 1 [file animals-11-02082-s001.zip › animals-1237941-supplementary.pdf]

Table S1. Effects of *Lawsonia intracellularis* vaccine on growth performance in weaned piglets.

Item	Treatments				SEM	*p*-Value
	**CON**	**LAW1**	**LAW2**	**LAW3**		
**Day 0 to 21**						
Initial BW, kg	6.47	6.47	6.47	6.02	0.49	0.888
Final BW, kg	10.94	11.18	12.72	11.64	0.99	0.603
ADG, g/d	212.86	224.29	297.62	267.62	32.77	0.280
ADFI, g/d	377.86	375.66	493.39	394.55	43.30	0.226
G:F. g/g	0.564	0.596	0.604	0.678	0.058	0.445
**Day 22 to 42**						
Initial BW, kg	10.94	11.18	12.72	11.64	0.99	0.603
Final BW, kg	24.13	25.43	26.69	25.53	1.42	0.662
ADG, g/d	628.10	678.60	665.24	661.43	34.02	0.753
ADFI, g/d	1030.36	1100.87	1094.41	1039.36	58.58	0.765
G:F, g/g	0.609	0.616	0.608	0.637	0.040	0.969
**Overall**						
Initial BW, kg	6.47	6.47	6.47	6.02	0.49	0.888
Final BW, kg	24.13	25.43	26.69	25.53	1.42	0.622
ADG, g/d	420.50	451.43	481.43	464.52	28.08	0.492
ADFI, g/d	704.11	738.26	793.90	717.26	43.18	0.497
G:F. g/g	0.597	0.611	0.607	0.648	0.033	0.717
**Fecal Score**						
Day 1 to 15	4.33	3.99	3.55	3.94	0.13	<0.05

Each value is the mean of 4 replicates (4 pigs/pen)

LAW1 – 0.5 dose, LAW2 – 1 dose, LAW3 – 2 dose

Fecal score 1 = Normal hard faeces, 2 = Slightly soft faeces, 3 = Soft, partially formed faeces, 4 = Loose, semi-liquid faeces, and 5 = Watery, mucous-like faeces [24]

Table S2. Number of 16S rRNA gene sequences before and after quality control in pigs orally challenged with *Lawsonia intracellularis* vaccine at week 0 and week 6.

Sample ID	Treatment	Week	Number of sequence reads		% QC-passed reads
			Pre-QC	Post-QC	
0.111	LAW1	W0	179570	69244	38.56
0.112	LAW1	W0	107246	28002	26.11
0.113	LAW1	W0	116581	22212	19.05
0.114	LAW1	W0	122964	34813	28.31
0.121	LAW1	W0	105427	29627	28.10
0.123	LAW1	W0	205386	72486	35.29
0.124	LAW1	W0	136822	24933	18.22
0.131	LAW1	W0	137248	46192	33.66
0.132	LAW1	W0	118818	17923	15.08
0.133	LAW1	W0	101498	21941	21.62
0.134	LAW1	W0	110578	27317	24.70
0.141	LAW1	W0	114739	23766	20.71
0.142	LAW1	W0	173256	69642	40.20
0.143	LAW1	W0	152686	54854	35.93
0.144	LAW1	W0	144074	46489	32.27
0.211	LAW2	W0	138926	53343	38.40
0.212	LAW2	W0	155603	58774	37.77
0.213	LAW2	W0	170361	70125	41.16
0.214	LAW2	W0	174161	71275	40.92
0.221	LAW2	W0	154093	62707	40.69
0.222	LAW2	W0	119086	27737	23.29
0.223	LAW2	W0	162870	59037	36.25
0.224	LAW2	W0	161945	58693	36.24
0.231	LAW2	W0	129567	30563	23.59
0.232	LAW2	W0	113419	26017	22.94

0.233	LAW2	W0	104922	16506	15.73
0.234	LAW2	W0	146816	62662	42.68
0.241	LAW2	W0	144866	52492	36.23
0.242	LAW2	W0	164114	62058	37.81
0.243	LAW2	W0	139905	48114	34.39
0.244	LAW2	W0	192833	49144	25.49
0.311	LAW3	W0	143520	41036	28.59
0.312	LAW3	W0	136272	31535	23.14
0.313	LAW3	W0	130172	33142	25.46
0.314	LAW3	W0	172585	73896	42.82
0.321	LAW3	W0	163701	64841	39.61
0.322	LAW3	W0	154201	34089	22.11
0.323	LAW3	W0	103954	29624	28.50
0.324	LAW3	W0	145139	53091	36.58
0.331	LAW3	W0	168844	49164	29.12
0.332	LAW3	W0	145540	50993	35.04
0.333	LAW3	W0	126018	24187	19.19
0.334	LAW3	W0	127701	33014	25.85
0.341	LAW3	W0	148862	42039	28.24
0.342	LAW3	W0	150983	33475	22.17
0.343	LAW3	W0	155883	59341	38.07
0.344	LAW3	W0	159311	39155	24.58
0.C11	CONT	W0	166548	61309	36.81
0.C12	CONT	W0	139644	54166	38.79
0.C13	CONT	W0	161935	59532	36.76
0.C14	CONT	W0	158305	63960	40.40
0.C21	CONT	W0	152518	58213	38.17
0.C22	CONT	W0	108560	36896	33.99
0.C23	CONT	W0	143510	52760	36.76
0.C24	CONT	W0	151261	60145	39.76

0.C31	CONT	W0	136986	35656	26.03
0.C32	CONT	W0	141231	53331	37.76
0.C33	CONT	W0	122522	37356	30.49
0.C34	CONT	W0	131563	46672	35.48
0.C41	CONT	W0	179678	71468	39.78
0.C42	CONT	W0	126711	38670	30.52
0.C43	CONT	W0	141252	56946	40.32
0.C44	CONT	W0	128549	26749	20.81
6.111	LAW1	W6	256609	93775	36.54
6.112	LAW1	W6	198547	63470	31.97
6.113	LAW1	W6	259813	119523	46.00
6.114	LAW1	W6	152740	55816	36.54
6.121	LAW1	W6	160485	47548	29.63
6.122	LAW1	W6	222984	73982	33.18
6.123	LAW1	W6	256306	95053	37.09
6.124	LAW1	W6	201268	68425	34.00
6.132	LAW1	W6	263900	90756	34.39
6.133	L AW1	W6	184455	53317	28.91
6.134	LAW1	W6	167998	45707	27.21
6.141	LAW1	W6	194367	82476	42.43
6.142	LAW1	W6	109197	41738	38.22
6.143	LAW1	W6	240154	83635	34.83
6.211	LAW2	W6	178666	71693	40.13
6.212	LAW2	W6	226323	73405	32.43
6.213	LAW2	W6	193555	56055	28.96
6.214	LAW2	W6	267063	98381	36.84
6.221	LAW2	W6	282347	110512	39.14
6.222	LAW2	W6	241049	90124	37.39
6.223	LAW2	W6	213356	68649	32.18
6.224	LAW2	W6	206341	68491	33.19

6.231	LAW2	W6	239847	75188	31.35
6.232	LAW2	W6	242858	89301	36.77
6.233	LAW2	W6	278483	101612	36.49
6.234	LAW2	W6	215165	70136	32.60
6.241	LAW2	W6	219404	81645	37.21
6.242	LAW2	W6	216435	106806	49.35
6.243	LAW2	W6	198089	81568	41.18
6.244	LAW2	W6	312006	125048	40.08
6.311	LAW3	W6	232374	61565	26.49
6.312	LAW3	W6	242946	86182	35.47
6.313	LAW3	W6	325783	127957	39.28
6.314	LAW3	W6	232366	83065	35.75
6.321	LAW3	W6	234540	97038	41.37
6.322	LAW3	W6	237266	109836	46.29
6.323	LAW3	W6	274739	113212	41.21
6.324	LAW3	W6	202872	81136	39.99
6.331	LAW3	W6	245005	96135	39.24
6.332	LAW3	W6	254266	115157	45.29
6.341	LAW3	W6	248644	89582	36.03
6.342	LAW3	W6	218454	93420	42.76
6.343	LAW3	W6	239066	92886	38.85
6.344	LAW3	W6	243389	116264	47.77
6.C11	CONT	W6	285043	108819	38.18
6.C12	CONT	W6	222342	75231	33.84
6.C13	CONT	W6	223204	70653	31.65
6.C14	CONT	W6	224888	73129	32.52
6.C21	CONT	W6	213215	76699	35.97
6.C22	CONT	W6	209984	63932	30.45
6.C23	CONT	W6	169061	46517	27.51
6.C24	CONT	W6	228458	82988	36.33

6.C31	CONT	W6	237012	101778	42.94
6.C32	CONT	W6	203569	61810	30.36
6.C33	CONT	W6	199076	58133	29.20
6.C34	CONT	W6	221142	81598	36.90
6.C41	CONT	W6	230461	88373	38.35
6.C42	CONT	W6	218451	97940	44.83
6.C43	CONT	W6	219696	102644	46.72
6.C44	CONT	W6	229970	97898	42.57

References

24 Wang, D.; Piao, X.S.; Zeng, Z.K.; Lu, T.; Zhang, Q.; Li, P.F.; Xue, L.F.; Kim, S.W.; Wang, D. Effects of Keratinase on Performance, Nutrient Utilization, Intestinal Morphology, Intestinal Ecology and Inflammatory Response of Weaned Piglets Fed Diets with Different Levels of Crude Protein. *Asian-Australas. J. Anim. Sci.* **2011**, *24*, 1718–1728, doi:10.5713/ajas.2011.11132.
